# Interaction of Hydrogen Sulfide with Nitric Oxide in the Cardiovascular System

**DOI:** 10.1155/2016/6904327

**Published:** 2015-11-10

**Authors:** B. V. Nagpure, Jin-Song Bian

**Affiliations:** Department of Pharmacology, Yong Loo Lin School of Medicine, National University of Singapore, Singapore 117597

## Abstract

Historically acknowledged as toxic gases, hydrogen sulfide (H_2_S) and nitric oxide (NO) are now recognized as the predominant members of a new family of signaling molecules, “gasotransmitters” in mammals. While H_2_S is biosynthesized by three constitutively expressed enzymes (CBS, CSE, and 3-MST) from L-cysteine and homocysteine, NO is generated endogenously from L-arginine by the action of various isoforms of NOS. Both gases have been transpired as the key and independent regulators of many physiological functions in mammalian cardiovascular, nervous, gastrointestinal, respiratory, and immune systems. The analogy between these two gasotransmitters is evident not only from their paracrine mode of signaling, but also from the identical and/or shared signaling transduction pathways. With the plethora of research in the pathophysiological role of gasotransmitters in various systems, the existence of interplay between these gases is being widely accepted. Chemical interaction between NO and H_2_S may generate nitroxyl (HNO), which plays a specific effective role within the cardiovascular system. In this review article, we have attempted to provide current understanding of the individual and interactive roles of H_2_S and NO signaling in mammalian cardiovascular system, focusing particularly on heart contractility, cardioprotection, vascular tone, angiogenesis, and oxidative stress.

## 1. Introduction

Endogenously produced hydrogen sulfide (H_2_S) is responsible for inducing variety of physiologically favorable effects in different mammalian body systems. It is the youngest member of “gasotransmitter” family, along with nitric oxide (NO) and carbon monoxide (CO) [1]. Considered as toxic and potentially lethal gases for centuries, they are now recognized by many researchers as the important cytoprotective endogenous modulators of many physiological functions.

Although NO was identified as a gas in late eighteenth century, its role as a biological agent was confirmed only in 1980 [2]. Its generation from NO synthase (NOS) and its action as a vasodilator were discovered a few years later in 1987 [3]. NO is formed from guanidine nitrogen of L-arginine by the action of 3 isoforms of NOS, namely, endothelial (eNOS), inducible (iNOS), and neuronal (nNOS) [4]. The identification of H_2_S as a toxic gas dates back even further than NO. The measurement of H_2_S revealed its existence in the brain [5]. This suggests its probable physiological importance. The gradual discoveries of cystathionine *β*-synthase (CBS) and cystathionine *γ*-lyase (CSE) as critical enzymes producing H_2_S [6] shed more light upon its signaling pathways and widespread physiological functions.

Being gaseous molecules and mediators, H_2_S and NO exhibit many common traits like the unique ability of free diffusion through cell membranes without the need of specific membrane receptors. Their endogenous enzymatic production is deftly regulated at many levels. Furthermore, they are also involved in modulation of many physiological processes in cardiovascular system (CVS) and central nervous system (CNS) [1]. While the individual signaling mechanisms mediated by H_2_S and NO in mammals are extensively studied, our understanding about the potential relationship between these two gasotransmitters is woefully incomplete. In 2009, first few definitive experimental evidences began to emerge voicing the probable “crosstalk” between H_2_S and NO [7]. Since then, it is now an established fact that these two gases influence each other at many levels from their biosynthesis to the various biological responses within cellular targets [8, 9]. The therapeutic potential of these gases is immense and thus being explored via many preclinical and clinical studies [10]. In this review article, we will focus on physiological and cellular functions mediated by H_2_S and NO in mammalian cardiovascular system. A prevailing understanding of the known and complex interplay between these gases and their signaling mechanisms would also be provided in the respective sections of the paper.

## 2. Synthesis and Metabolism of H_**2**_S in Mammalian Cells

**Figure 1:**
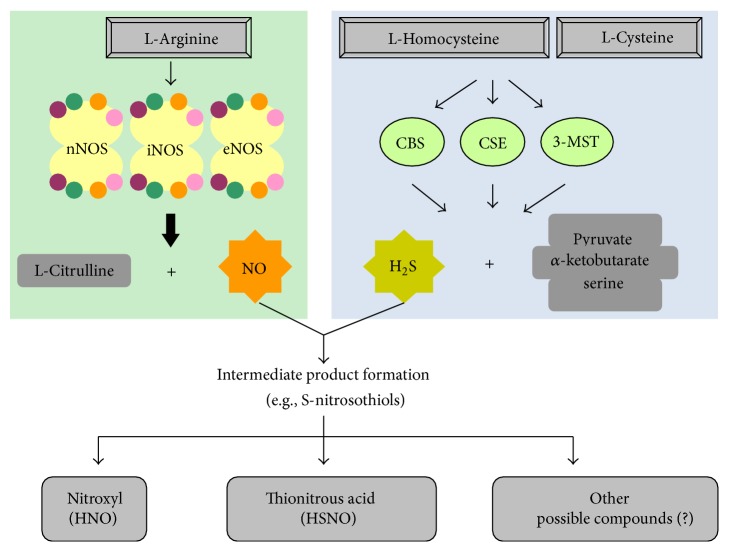
Biosynthesis of NO and H_2_S. NO is synthesized by three different isoforms of NOS, namely, nNOS, eNOS, and iNOS. These isoforms are almost identical in structure and functions. A functional NOS is made up of two identical monomers, each with four cosubstrates (NADPH, FAD, FMN, and BH_4_). L-Arginine is oxidized into L-citrulline along with the production of NO. H_2_S is produced by the catalytic action of 3 different enzymes, CBS, CSE, and 3-MST. Each enzyme is expressed in organ-specific manner and catalyses the production of H_2_S by oxidizing L-homocysteine and L-cysteine. Pyruvate, *α*-ketobutyrate and serine are produced as bi-products. The interaction between these two gases can give rise to production of few intermediate products (e.g., S-nitrosothiols). As explained in this review article later, nitroxyl (HNO), thionitrous acid (HSNO), and other possible unknown compounds have been shown to induce some interesting effect in cardiovascular system.

A major contribution in the endogenous production of H_2_S is offered by two pyridoxal-5′-phosphate- (PLP-) dependent enzymes, namely, CSE and CBS. They utilize L-cysteine or homocysteine as substrates [11]. Recently, however, a study reported that 3-mercaptopyruvate sulfurtransferase (3-MST) acts together with cysteine aminotransferase (CAT) to generate H_2_S in the brain (Figure 1). They also suggested that 3-MST and CAT are primarily involved in the neuronal production of H_2_S [12]. While CBS and CSE are mainly cytosolic, 3-MST is preferably expressed in mitochondria [13]. Furthermore, their distribution is highly tissue specific. CBS is primarily detected in neurons and astrocytes of CNS [14], whereas CSE is located in the CVS, especially the myocardial cells [15] and vascular smooth muscle cells [16]. The localization of CSE in endothelial cells (EC) is a bit controversial. A few research groups have detected its expression in the ECs [17, 18] while others have not reported as such [19, 20].

H_2_S production by CBS involves the condensation reaction between homocysteine with L-cysteine to produce cystathionine and H_2_S [21]. CSE catalyzes the conversion reaction of L-cysteine into thiocysteine and pyruvate. The thiocysteine thus generated is lysed to form cysteine and H_2_S [22]. CAT, on the other hand, catalyzes the synthesis of 3-mercaptopyruvate from L-cysteine and *α*-ketoglutarate. 3-MST then desulfurates 3-mercaptopyruvate to generate thiosulfate. Later, H_2_S is generated by reduction of thiosulfate [23]. Recently, Shibuya et al. identified a novel pathway of H_2_S production specifically in kidney and the cerebellum region of the brain. H_2_S can be generated from D-cysteine by activation of 3-MST and D-amino acid oxidase [24]. The intracellular storage forms of H_2_S have also been identified. Acid-labile sulfur is mainly located in the iron-sulfur cluster of mitochondria. Measured in the form of sulfide, acid-labile sulfur has been detected in brains of rats, bovines, and humans. It releases H_2_S only in acidic microenvironment (pH = 5.4) [19]. Due to the highly unstable nature of iron-sulfur clusters, H_2_S is readily released when needed [20]. Bound sulfane sulfur, which is presented in cytosolic region, contains divalent sulfur bond (e.g., persulfide form). It releases H_2_S under basic conditions (pH 8.4) [20]. It is speculated that H_2_S produced by 3-MST/CAT enzymatic pathway is stored in the bound sulfane sulfur form as lesser amount of bound sulfane sulfur has been detected in cells without 3-MST/CAT [15].

Under the physiological conditions, H_2_S is quickly eliminated by various routes. Mitochondrial oxidation of deprotonated HS^−^ results into thiosulphate, which is further converted into sulfite and eventually sulfate. Sulfate production is the primary fate of H_2_S metabolism [25]. H_2_S also undergoes cytosolic methylation by thiol S-methyltransferase to produce dimethylsulfide and methanethiol. H_2_S has high affinity towards hemoglobin. Thus, H_2_S binds to hemoglobin producing sulfhemoglobin [19].

## 3. Synthesis and Metabolism of NO in Mammalian Cells

Three different isoforms of the enzyme NOS produce NO in mammals. They are commonly known as neuronal nNOS (NOS I), inducible iNOS (NOS II), and endothelial eNOS (NOS III). Although genetically distinct, all three isoforms form NO from L-arginine with the help of two cosubstrates, namely, molecular O_2_ and nicotinamide-adenine-dinucleotide phosphate (NADPH). The biosynthesis also requires various cofactors like flavin mononucleotide (FMN), flavin adenine dinucleotide (FAD), and tetrahydrobiopterin (BH_4_) [4]. In a typical biosynthesis reaction, catalytically active NOS transfers electrons from NADPH to the heme, via FAD and FMS [26]. Calmodulin is bound to the reductase domains of monomers and it helps in facilitating the transfer of electrons [27]. These transferred electrons facilitate binding of molecular O_2_ to the ferrous form by reducing iron in the heme. The ferrous form is then combined to L-arginine to synthesize L-citrulline and NO [27, 28] (Figure 1). NO, thus generated, activates number of downstream secondary signaling pathways like soluble guanylyl cyclase activation which results into cGMP formation [29].

Besides CNS, nNOS is also identified in autonomic nerves of smooth muscles in blood vessels, gastrointestinal tract, respiratory tract, and genitourinary tract [30]. The expression of iNOS is identified in many immunological cell types such as macrophages [31] and neutrophils [32]. The third form, eNOS, is mainly expressed in endothelial cells but also present in other cell types such as cardiomyocytes, hepatocytes, intestinal cells, platelets, neurons, and astrocytes [33].

nNOS is primarily activated by glutamate acting on NMDA receptors. The enzyme activity is regulated by glutamate-induced rise in intracellular calcium ([Ca^2+^]_i_) level and its interaction with calmodulin. Unlike nNOS, iNOS is neither affected by [Ca^2+^]_i_ levels nor dependent on the presence of cosubstrate NADPH and cofactor BH_4_ [34]. Its activity is stimulated by exposure to pathological insults, especially bacterial endotoxins and proinflammatory cytokines such as TNF-*α* and interleukins. The activation of eNOS is triggered by increased [Ca^2+^]_i_, which in turn is elevated by phosphoinositide secondary signaling pathway. Similar to nNOS, eNOS activity is Ca^2+^ dependent and is regulated by calmodulin [30].

The nitrite (NO_2_^−^) and nitrate (NO_3_^−^), collectively known as NO_*x*_, are the end products of endogenous NO metabolism in the mammalian cells [35]. They are also recycled physiologically to generate NO and other nitrogen oxides [36]. Recently, they have been acknowledged as “storage pools” of NO in mammalian tissues, complementing the NOS-dependent pathway of NO biosynthesis [35]. In another proposed mechanism of NO storage, reduced glutathione (GSH) is nitrosylised to generate S-nitroso-L-glutathione (GSNO) [37]. NO stored in the form of GSNO can be released by the action of many enzymes such as GSH peroxidase [38] and thioredoxin reductase [39].

Physiologically, endogenously generated NO is rapidly metabolized. It diffuses through lumen of blood vessels and intracellular compartments to react with hemoglobin. This elimination pathway leading to the formation of nitrates is considered as the major mechanism of NO catabolism [40]. In another mechanism, NO is oxidized in blood forming nitrites which react with hemoglobin producing nitrates [37]. As discussed before in this paper, excessive production of NO can be detrimental to the cells. This excessive NO reacts with bicarbonate to produce nitrosoperoxycarbonate (ONOOCO_2_^−^) and thus scavenged from the body [41].

## 4. Biochemistry of NO-H_**2**_S Interaction

The biological and chemical reactivities of H_2_S have been discussed thoroughly in some excellent review papers previously [42, 43]. After dissolving in water, H_2_S dissociates in H^+^, HS^−^, and S^2−^. The anionic form HS^−^ contributes to the major share while S^2−^ exists in a very small amount at the physiological pH [44]. The H_2_S/HS^−^ form is a strong reducing agent, which is capable of reducing many organic substrates including NO and its oxidized forms. H_2_S can form chemical complexes with nitrate, nitrite, S-nitrosothiols, and peroxinitrates [45]. In a previous work, Whiteman et al. demonstrated that mixture of various NO donors and NaHS (H_2_S donor) forms a novel species known as nitrosothiols [46]. The study revealed that the addition of H_2_S to various NO donors not only inhibits the release of NO, but also alters the expected NO-based biological function. Their mechanism of formation is elusive but the direct reaction between H_2_S and NO can be ruled out as H_2_S/HS^−^ exists in diamagnetic acid/base pair while NO exhibits paramagnetic nature at the physiological pH. The aerobic conditions maintained during these experiments might be responsible for NO oxidation leading to the formation of nitrosating species.

**Figure 2:**
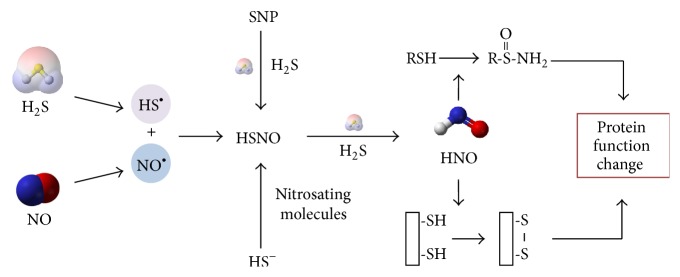
Simplifying depiction of chemical interaction between H_2_S and NO. The radical of H_2_S (HS^•^) reacts with that of NO (NO^•^) to generate thionitrous acid (HSNO). In an alternate way, HSNO can be produced by hydrosulfide ion (HS^−^), an anionic form of H_2_S, after its reaction with different nitrosating biomolecules. Sodium nitroprusside can also produce HSNO by reacting with H_2_S. In a further step of reaction, HSNO is acted upon by H_2_S to produce HNO. HNO may modify the functions of proteins by converting their reactive thiols (thiolates) in cysteine residues to N-hydroxysulfenamide (RSNHOH) or forming disulfide bond between two thiol groups in the near vicinity.

H_2_S can reduce oxidized NO forms leading to the formation of HSNO as an intermediate. Further reduction and direct displacement of HSNO by H_2_S results in the formation of yet another intermediate product, nitroxyl (HNO) [47] (Figure 2). HNO produces chemical and physiological functions different from NO [48] and H_2_S [9, 49]. HNO is highly redox-sensitive and therefore regulates protein functions through “redox switches” [50, 51]. HNO can react with thiol groups in the cysteine residues to form N-hydroxysulfenamide (RSNHOH) [52] or helps to form a reversible disulfide bond if there are two thiols residing in the near vicinity [53]. These modifications may induce conformational change and therefore the functions of the targeted proteins (Figure 2). The pharmacological effects of HNO donors have already brought attention of many research groups towards their potential therapeutic value against many cardiac ailments such as congestive cardiac failure. There are several types of HNO releasing compounds. The most commonly used one is Angeli's salt (Na_2_ N_2_O_3_). Other donors include Piloty's acid (PhSO_2_NHOH) and its derivatives, isopropylamine-NO^•^ (IPA/NO), and acyloxy nitroso compounds such as 1-nitrosocyclohexyl acetate (NCA, also known as the “blue compound”) [54]. In the upcoming sections of this paper, we have discussed the effects of HNO on various aspects of cardiovascular physiology.

## 5. H_**2**_S-NO Interaction in Cardiovascular System

### 5.1. Role of H_2_S-NO Interaction in the Regulation of Heart Contractility

NO (both exogenous and endogenous) has concentration-dependent bimodal action on basal contractile state of cardiomyocytes. At low concentrations, NO exerts positive inotropic action [55]. The low NO levels activate adenylyl cyclase (AC) and downstream cAMP dependent signaling pathway [56]. Thus activated protein kinase A (PKA) phosphorylates voltage-dependent calcium channels and opens sarcoplasmic ryanodine receptors (Ry/R) [57]. The resultant increase in [Ca^2+^]_i_ is mainly responsible for positive inotropic action. On the other hand, the negative inotropic effect by higher NO concentration is mediated chiefly through cGMP dependent pathway. The increased intracellular cGMP is further shown to downregulate myofilament calcium sensitivity increasing cardiac relaxation [58]. The cGMP regulator phosphodiesterase 5A (PDE5A) is shown to modulate cardiac *β*-adrenergic stimulation in an eNOS dependent manner [59]. It is interesting to know that the mechanisms of action of NO depend upon the origin of endogenous NO as well. nNOS-derived NO has been demonstrated to upregulate cardiac contractility by direct protein S-nitrosylation of Ry/R receptors [60].

NaHS also produces a negative inotropic effect on cardiomyocytes by suppression of opening of K_ATP_ channels [15, 61], blockade of L-type calcium channels [62], and suppression of cAMP/PKA pathway [63]. Accumulating evidences suggest that there is a cross-talk between H_2_S and NO in the heart. H_2_S may directly interact with NO during pathological situations like oxidative stress and alter cardiac functions. We were among the first groups to observe that H_2_S reversed the negative inotropic and lusitropic effects of NO. Mixing NO donors (SNP, SIN-1, or SNAP) with NaHS produces an opposing effect on heart contractility as compared with either gas alone. To explain this phenomenon, we proposed the formation of new thiol sensitive molecule as they found that thiols abolished the effects of the mixture of NO and H_2_S in their experimental setup [9, 49]. It is also possible that H_2_S reacts with either oxidized forms of NO (e.g., NO^•^) or nitrogen species (ONOO^−^) through HS^−^ in the presence of cellular oxidants for example, molecular oxygen, ROS (e.g., H_2_O_2_), and oxidases. This process may generate new molecules like nitrosothiol, thionitrous acid (HSNO), or HNO [64]. Due to the strong reducing capability of H_2_S [1, 65, 66], Yong et al. proposed that HNO could be one of the possible candidates [49]. This hypothesis was further confirmed by another group who studied the production of intracellular HNO in cells treated with nitrite/H_2_S reaction mixture with an HNO sensor (CuBOT1) [67]. The similar results were observed when sodium nitroprusside (SNP) was used as a NO donor [68, 69]. The interaction of H_2_S with NO and the resultant synthesis of thiol-sensitive compounds may also provide the justification behind the elusive bimodal effect of NO on cardiac contractility as mentioned in the beginning of this section.

Although the mechanisms for the positive inotropic effect of HNO are still not well understood, it is now believed that it is mediated by a *β*-adrenoceptor independent pathway [70, 71]. Inhibition of cAMP/PKA and cGMP/PKG had no significant impact on its inotropic effect [72]. In fact, the redox dependent mechanism is important for the positive inotropic effect of HNO. HNO can enhance the myofilament calcium sensitivity through formation of an actin–TM heterodimer. With mass spectrometry (MS) and a modified biotin switch assay, Gao et al. even found out the four cysteine residues in myofilament modified by HNO [8]. HNO can also modulate the thiol groups in EC-coupling proteins and regulate the functions of these proteins. For instance, HNO modulates SERCA2a/phospholamban (PLN) interaction and therefore stimulates SR function [57]. More experiments revealed PLN is important in the HNO inotropy/lusitropy, as mutation of the three cysteine residues in PLN transmembrane domain abolished the effect of HNO [73]. Tocchetti et al. showed that the effect of HNO was from a direct interaction of HNO with the sarcoplasmic reticulum Ca^2+^ pump and the ryanodine receptor 2, leading to increased Ca^2+^ uptake and release from the sarcoplasmic reticulum [72].

**Figure 3:**
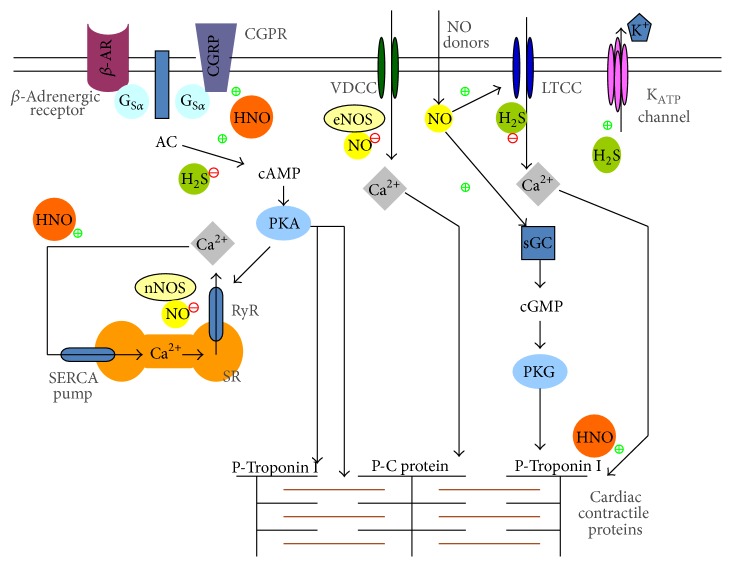
Effects of NO, H_2_S, and HNO on heart contractile function. The negative inotropic effect of NO is mediated mainly by cGMP-PKG pathway in CVS. Exogenous NO is believed to act via direct phosphorylation of LTCC and cardiac contractile proteins such as troponin 1. The effect of endogenous NO depends on the source. eNOS-generated NO acts via cGMP dependent pathway. nNOS-generated NO S-nitrosylates ryanodine receptors of sarcoplasmic reticulum. H_2_S also exerts negative effect on cardiac contractility via (1) opening of K_ATP_ channels, (2) blockade of LTCC, and (3) inhibition of cAMP signaling pathway. Interestingly, the intermediate product, nitroxyl (HNO), produces positive inotropic effect. The possible underlying mechanisms of action include stimulation of calcitonin gene-related peptide signaling and enhancing cardiac sarcoplasmic reticulum Ca^2+^ cycling.

In addition, Paolocci et al. reported that the positive inotropic signaling was mediated by calcitonin gene-related peptide (CGRP), as treatment with the selective CGRP-receptor antagonist CGRP (8–37) prevented this effect [71]. However, this finding was later disproved as positive inotropic effects of CGRP were found to be mere sympathostimulatory in nature and downregulated by *β*-adrenoceptor blockers [74]. Nonetheless, the positive inotropic/lusitropic action of HNO render it to be an attractive addition to the current therapeutic armamentarium for treating patients with acutely decompensated congestive heart failure [75] (Figure 3).

### 5.2. Role of H_2_S-NO Interaction in the Cardioprotection

Myocardial ischemia occurs when cardiac myocytes are insufficiently provided with the oxygenated blood via coronary arteries, resulting in cardiovascular morbidity and mortality [76]. Ischemic injury is a complex process involving the action and interaction of many factors. NO is one of these factors to protect heart against ischemic injury. The studies conducted in eNOS deficient (eNOS^−/−^) mice [77] and eNOS overexpressing mice [78, 79] have concluded that eNOS-derived NO is a strong endogenous cardioprotective agent against cardiovascular pathologies including ischemia-reperfusion (I/R) injury and congestive cardiac failure. The administration of NO donors also has similar protective effects in I/R injury and other heart diseases in humans and other mammals [80–82]. The studies have revealed different possible underlying mechanisms including activation of sGC/cGMP/PKG signaling pathway [83], activation of subcellular K_ATP_ channels [84, 85], and Ca^2+^ influx inhibition [86].

Similarly, the cardioprotective effects of H_2_S also involve multiple mechanisms (Figure 3). This was described in detail in our previous review article [64]. Downregulation of endogenous H_2_S production was found to increase myocardial infarct size, suggesting an important role of endogenous H_2_S in maintaining the normal heart function [87]. In different animal models, H_2_S was shown to protect heart against I/R injury via diverse mechanisms. Zhang et al. reported that H_2_S stimulated opening of K_ATP_ channels in cardiomyocytes [88]. The contribution of antiapoptotic signaling activation was demonstrated by the modulation of proteins expression including Beclin-1 [89], Bcl-2, Bax, caspase 3 [90], and HSP-90 [91]. H_2_S is also known to preserve mitochondrial functions by modulating cellular respiration [92]. We and other groups revealed that the cardioprotective effect of H_2_S preconditioning involves the activation of PKC and sarcolemmal K_ATP_ channels, Akt, and eNOS pathways [93–96].

H_2_S and NO may act in concert to protect the heart against ischemic injury. Inhibition of NO production with L-NAME, a nonselective inhibitor of NO synthases, significantly attenuated the cardioprotective effects of H_2_S preconditioning [97]. Administration of NaHS alleviated isoproterenol-induced toxic cardiomyopathy through elevation of myocardial and serum NO levels [98]. H_2_S may regulate NO production through modulation of eNOS and iNOS expression and activity. We showed previously that H_2_S pretreatment activates eNOS pathway to confer protective effect against ischemic injury [93]. In an interesting study conducted in human umbilical vein endothelial cells (HUVECs-926), both eNOS phosphorylation and NO production were upregulated upon treatment with NaHS [99]. Moreover, malfunction of eNOS and reduced NO level were also found in CSE knockout mice. This contributes to the impaired heart function during I/R injury [100]. However, some conflicting effects were also reported. The data collected from rat and mouse aortic rings demonstrated that H_2_S directly inhibited recombinant bovine eNOS activity [101]. In yet another study, both exogenous and endogenous H_2_S inhibited eNOS transcription and activity [102]. Thus it is highly possible that the nature of effect of H_2_S on eNOS is dependent on many factors including H_2_S concentration and experimental setup.

Overexpression of iNOS and the subsequent excessive formation of NO may cause cytotoxic effects and exacerbate myocardial injury [103]. Inhibition of iNOS may produce beneficial effects in heart [104]. Apart from regulation of eNOS, H_2_S also modulates iNOS expression. Hua et al. found that H_2_S protected heart against CVB3-induced mice myocarditis through suppression of iNOS expression and the subsequent HO-1 pathway [105]. Taken together, NO is an important player in the cardioprotection induced by H_2_S, despite different mechanisms that may be involved in various pathological situations.

In contrast to the intensive investigation on the effect of H_2_S on NO generation, little is known about the effect of NO on H_2_S production. A previous study showed that exogenous application of an NO donor, sodium nitroprusside, and upregulated the expression of CBS and CSE, culminating in augmented H_2_S production in rat tissues [106]. These data suggest that H_2_S and NO may influence the production of each other by altering their generating abilities during ischemic situations.

**Figure 4:**
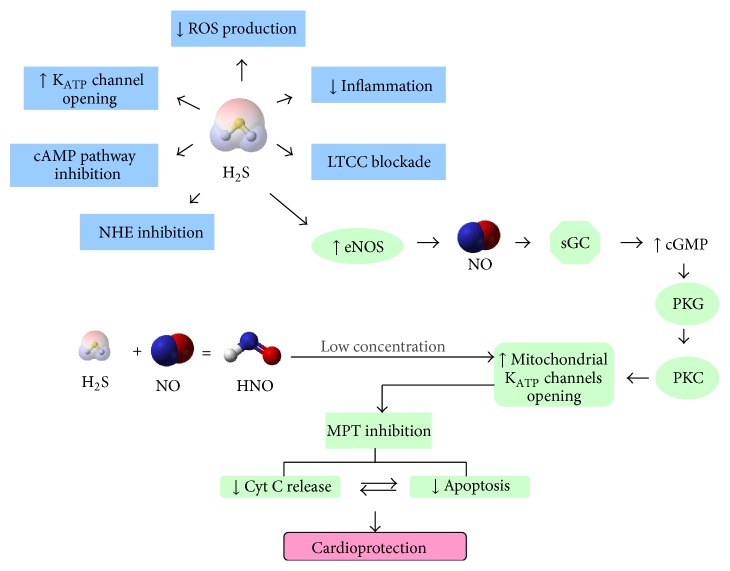
Cardioprotective effects of NO, H_2_S, and HNO. There are multiple underlying mechanisms for cardioprotective effect of H_2_S. It has been demonstrated to induce K_ATP_ channel opening, abolish inflammation and oxidative stress by inhibiting ROS production, block LTCC and intracellular cAMP signaling pathway, and inhibit Na^+^/H^+^ exchanger activity. Furthermore, NO also plays an important role in the cardioprotective effect of H_2_S. Endogenous NO generated from eNOS in endothelial cells initiates the sGC-cGMP-PKG cascade to increase openings of subcellular (mitochondrial) K_ATP_ channels. The resultant inhibition of mitochondrial permeability transition (MPT) is responsible for decreased cytochrome C release and apoptosis in cardiomyocytes. HNO, depending on the concentration, can be either cardioprotective or cardiotoxic. At low concentration, just like NO, HNO also stimulates K_ATP_ channels opening in mitochondria.

However, the role of HNO, the direct interaction product from these two gases, in ischemic reperfusion injury is still debated. Preconditioning with HNO also grants a protection similar to that afforded by classical ischemic preconditioning [107]. This protective effect was not from NO, as it cannot be achieved with equimolar amounts of the NO donors. The mechanisms underlying HNO-induced cardioprotection may involve mitochondrial K_ATP_ channel (mK_ATP_) [108] (Figure 4). However, it is also worth noting that higher concentration perfusion of HNO may also produce detrimental effects during ischemic reperfusion caused by recruitment of neutrophils [109].

### 5.3. Role of H_2_S-NO Interaction in the Maintenance of Vascular Tone

The identification of NO as an endothelium derived relaxing factor [3] is a milestone in the field of gasotransmitters biology research. NO is now established as an important regulator of vascular tone. Physiologically, NO is a powerful vasodilator exerting its effect on various arteries, resistance vessels, and veins. The underlying signaling pathway is mainly cGMP dependent [110]. NO can also mediate vasodilation in a cGMP independent manner [111, 112]. S-Nitrosohemoglobin formed by S-nitrosylation of Cys93 of the hemoglobin *β* subunit has been demonstrated to moderate hypoxic vasodilation [113, 114].

**Figure 5:**
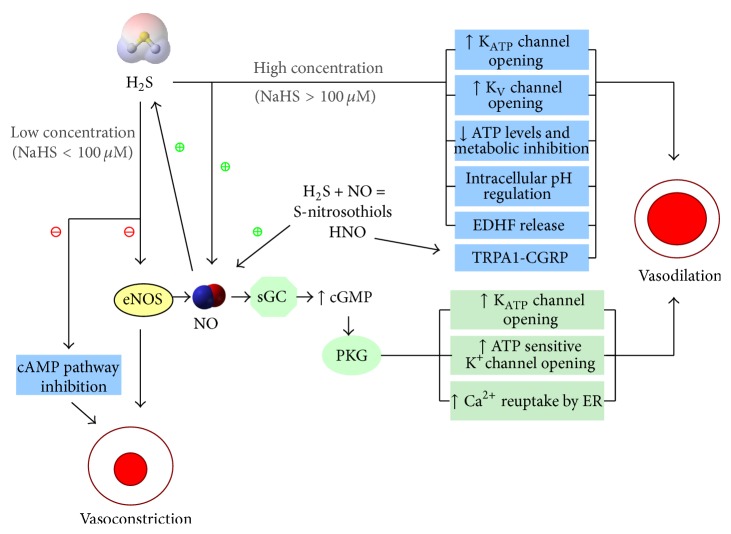
Regulatory effect of NO, H_2_S, and S-nitrosothiols on vascular tone. Depending upon the concentration, H_2_S exerts biphasic response on vascular tone. At high concentration (NaHS > 100 *μ*M), H_2_S acts as a potent vasodilator by opening of K_ATP_ and K_V_ channels, downregulating ATP levels and cellular metabolism, regulating intracellular pH and release of endothelium-derived hyperpolarizing factor (EDHF). At low concentration (NaHS < 100 *μ*M), however, H_2_S acts as a vasoconstrictor by inhibiting eNOS-derived NO production and intracellular cAMP pathway. NO, by itself, is a strong vasodilator. Acting via cGMP-PKG pathway, it stimulates opening of K_ATP_ and ATP-sensitive potassium channels. It also increases Ca^2+^ reuptake by endoplasmic reticulum and thus decreases intracellular Ca^2+^ levels resulting in less contraction. NO signaling is also stimulated by high concentration of H_2_S, contributing to its vasodilatory effect. S-nitrothiols, derived from interplay between NO and H_2_S, act as vasodilators mainly via NO signaling.

H_2_S has a biphasic effect on vascular tone in the cardiovascular system by mediating both vasorelaxation and vasoconstriction (Figure 5). Exogenously applied H_2_S in higher concentrations (NaHS > 100 *μ*M) relaxes vascular smooth muscles. It is suggested that the vasodilatory effect of endogenous H_2_S is mainly responsible for the maintenance of basal tone in vasculature which in turn controls physiological blood pressure [115]. H_2_S targets K_ATP_ channels to produce its vasodilatory effect [16, 115]. Additional mechanisms such as involvement of the Ca^2+^ channels [116], Cl^−^/HCO_3_^−^ exchanger [117], and metabolic inhibition [118] are required for the vasorelaxant effects of H_2_S. Interestingly, Ali et al. demonstrated the reversal of relaxant effect of endothelium/NO-dependent vasodilators (ACh and Histamine) by the treatment of H_2_S in lower concentration (NaHS < 100 *μ*M) [119]. This finding is in accordance with the previous results, where NaHS at concentration of 30 *μ*M induced a strong vasoconstrictive effect by itself. The mechanisms underlying the vasoconstrictive effects of low concentration of H_2_S involve downregulation of endothelial NOS, decrease of intracellular cAMP level in smooth muscle cells, and production of ROS. This was discussed in details in our previous review [64].

Various experimental studies provided evidence for the interaction between H_2_S and NO and the vasoregulatory role of this interaction. The first report of summation effect between H_2_S and NO on vasorelaxation came from the findings of Hosoki et al. which demonstrated that H_2_S can induce stronger relaxation effect in the presence of a NO donor [120]. Furthermore, pharmacological blockade of endogenous NO production or physical removal of the endothelium, attenuated H_2_S-induced relaxation [16]. These data suggest that the vasorelaxant effect of H_2_S is mediated by NO. The interplay between these two gases is different for the observed effect of vasoconstriction. Zhao and Wang found that H_2_S inhibited SNP-induced vasorelaxation [116]. In line with this finding, Ali et al. found that a mixture of NO and H_2_S reduced the extent of vasorelaxation compared to the relaxation with NO alone, implying the regulation of availability of NO by H_2_S. Interestingly, H_2_S only induced vasoconstriction in endothelium-intact vessels but not in endothelium-denuded vessels.

The contractile effect of H_2_S is therefore not a direct action on vascular smooth muscle cells but an indirect effect involving endothelial cells. Furthermore, they demonstrated that NaHS, in a dose-dependent manner, significantly downregulated vasorelaxant effect induced by chemically different NO donor molecules (e.g., SNP, SNAP). Similarly, NaHS reversed vasorelaxation induced by endogenous NO (from vascular endothelial cells) in a concentration dependent manner. This indicates that H_2_S may induce vasoconstriction via direct quenching of NO. Interestingly, this group also hypothesized the formation of a new compound, nitrosothiol. Since copper sulfate, which converts nitrosothiol to nitrite and nitrates, prevented the contractile of aortic rings without influencing the vasorelaxant effect of NaHS, the generation of nitrosothiols was proved. This nitrosothiol molecule might have contributed to the modulatory effect of H_2_S on vascular tone [119]. Similarly, we found that H_2_S may also stimulate anion exchanger-2 activity which transports HCO_3_^−^ in exchange of O_2_^−^ to inactivate NO and thus inducing stronger vasoconstriction. In extracellular space, O_2_^−^ reacts with NO to form ONOO^−^ [121]. Since NO uptake by SMC is positively dependent on the level of intracellular O_2_^−^ in SMC [122], the depletion of intracellular O_2_^−^ may further inhibit NO uptake in SMC. These findings indicate that H_2_S may induce vasoconstriction via inactivation of NO.

Recently, Berenyiova et al. found that of the interaction of sodium sulfide (Na_2_S) and S-nitrosoglutathione (GSNO) relaxed precontracted isolated rings of rat thoracic aorta and mesenteric artery with a much stronger potency than any of these two chemicals alone. They claimed that the formation of nitroxyl (HNO) is responsible for the pronounced relaxation induced by the sulfide/GSNO cross-talk [123].

HNO is produced endogenously in vascular tissue [124–126]. It induces vasodilatory effect via multiple mechanisms. Previous reports showed that HNO may dilate vascular vessels as an endothelium-derived relaxing and hyperpolarizing factor [127, 128], via activation of a cGMP-dependent pathway [129] and via activation of TRPA1 receptor channels of trigeminal fibres inducing CGRP release [130]. Interestingly, not like NO, HNO does not develop tolerance in human blood vessels [129].

In addition to the direct interaction, H_2_S and NO are also known to affect mutual production. NO can increase H_2_S production in the normal vascular tissues. Incubation with NO donors increased H_2_S production rate in the rat vascular tissues [16, 106]. In pulmonary hypertension, higher H_2_S production and upregulated CSE level were found in the presence of L-arginine [131]. On the other hand, H_2_S may downregulate the aortic L-arginine/NO pathway [101, 102, 121]. H_2_S inhibited recombinant eNOS activity and thus reduced NO synthesis in the endothelium [101]. In aortic tissues, Geng et al. also reported that H_2_S suppressed NO production by inhibition of eNOS transcription, abundance. and activity [102]. Coletta et al. determined the cooperative effect of H_2_S and NO by silencing CSE. It attenuated the NO donor induced cGMP accumulation and vasodilator-stimulated phosphoprotein (VASP) [132]. In a recent study, Eberhardt et al. showed that HNO formed from H_2_S and NO activated transient receptor potential channel A1 (TRPA1). The sensory chemoreceptor channel TRPA1 was activated via formation of amino-terminal disulphide bonds, which resulted in sustained Ca^2+^ influx. Consequently, calcitonin gene-related peptide (CGRP) was released inducing potent local and systemic vasodilation [133]. Thus it can be proposed that the H_2_S and NO homeostasis is of the prime importance in maintaining vascular tone.

Short term application of exogenous H_2_S reduced NO formation in cultured human umbilical vein endothelial cells through suppression of protein expression of eNOS but not those of nNOS and iNOS [102]. However, Huang et al. found that treatment with NaHS or H_2_S releasing donor, ACS14, for 24 h attenuated the increase in iNOS expression caused by high glucose (25 mM). This is similar to the inhibitory effect of H_2_S on iNOS expression in heart [134]. These data suggest that H_2_S may regulate iNOS expression in a time-dependent manner.

### 5.4. Role of H_2_S-NO Interaction in Angiogenesis

The formation of new blood vessels from preexisting vasculature through process of angiogenesis is the means by which cells can meet an elevated need of metabolites and in pathological conditions such as ischemia. Endothelial cells (ECs) play a pivotal role in the process by migrating towards and proliferating at the site of angiogenesis [135, 136].

Accumulating evidences suggest that gasotransmitters NO and H_2_S are important factors to influence ECs and angiogenesis [8]. The relationship between NO and neovascularization is very well established [137] and found to involve cGMP transduction pathway [8]. Many angiogenic growth factors such as VEGF and basic fibroblast growth factor enhance eNOS expression and stimulate its activity to produce NO [138]. Cai et al. observed that NaHS stimulated the* in vitro* parameters of angiogenesis such as cell growth, migration, scratched wound healing, and tube-like structure formation in cultured RF/6A endothelial cells [139]. It was speculated that H_2_S exerts its effects on ECs through K_ATP_ channels that in turn facilitate activation of MAPK pathways, leading to new blood vessel formation [140].

The signaling mechanisms of H_2_S and NO are not mutually exclusive for angiogenesis. In an exhaustive study conducted by Coletta et al., PKG was concluded to be a converging point for the secondary signaling mechanisms of H_2_S and NO [132]. In accordance with the previous results [141], this group found that the exogenous application of H_2_S decreased cGMP degradation by inhibiting PDE5A. This effect on intracellular cGMP is aided and abetted by NO which activated sGC to stimulate the production of intracellular cGMP. As mentioned previously, H_2_S stimulates Akt to induce its angiogenic effect. The stimulation of Akt in turn induces eNOS phosphorylation [142]. This particular response suggests that H_2_S influences eNOS activity. Very few studies have addressed the role of HNO in angiogenesis. The first strong indication for the probable antiangiogenic role of HNO came from the studies conducted in animal models of neointimal hyperplasia. It was observed that inhibition of EC proliferation was partly responsible for inhibitory effects of IPA/NO on neointimal hyperplasia. It should be noted that either IPA/NO itself or products of IPA/NO decomposition could have caused these effects [143]. While working on* in vitro* and* in vivo* models of breast cancer, Norris et al. found that HNO treatment not only reduced blood vessel density but also downregulated angiogenesis. They observed lower levels of circulating serum VEGF and HIF-1*α*, both of which are potent proangiogenic factors [144].

## 6. Role of H_**2**_S-NO Interaction in Oxidative Stress in CVS

Obesity, hypertension, and aging are few distinct causative factor for cardiovascular diseases. They are accompanied by oxidative stress, which is the result of imbalance between ROS generating and ROS-scavenging systems [145–147]. It is now a well-established fact that ROS generation is ramped up in heart [134] and blood vessels [135] during cardiovascular pathologies. Oxidative stress is a result of excessive production of ROS like O_2_^−^, ^•^HO, H_2_O_2_, NO, ONOO^−^, and HClO, mainly as a byproducts of cellular aerobic metabolism. The action of certain enzymes like NADPH oxidase and NOS is of also crucial [137]. NADPH oxidase activity and mitochondrial electron transport chain are mainly responsible for ROS production in aging heart [138] and vasculature [139]. Increased ROS generation has many harmful consequences like stimulation of inflammatory response, apoptosis, and ER stress culminating into cellular damage [140].

H_2_S is a well-known antioxidant [141] and it has been shown to protect vascular endothelial function under conditions of acute oxidative stress by directly scavenging O_2_^−^ and downregulating vascular NADPH oxidase-derived O_2_^−^ production [142]. It has been reported that NO downregulates NADPH oxidase-dependent superoxide production in human endothelial cells by S-nitrosylation of p47phox subunit [148]. The chemical properties of HNO suggest that it can act as a potent antioxidant [149] as well. The low dissociation energy of H-NO bond [150] makes HNO a strong reducing agent. Thus, HNO is speculated to quench reactive intermediate products produced during radical oxidation processes like lipid peroxidation [149]. It should also be noted that oxidation of HNO leads to production of NO, which itself is an antioxidant in nature [151]. Furthermore, HNO is demonstrated to have an effect on cGMP-dependent signaling pathway, which incidentally is a potent ROS-suppressing mechanism in the heart. The results of a study conducted by Lin et al. show that HNO suppresses NADPH oxidase by upregulating sGC and cGMP signaling in neonatal rat cardiomyocytes [152]. Interestingly, Miller et al. latest work revealed the sGC-cGMP-independent mechanism of action of HNO. They observed that HNO donors directly inhibited the activity of NADPH oxidase (vascular Nox2) in mouse cerebral arteries. They also proposed that HNO modifies reactive cysteine thiols in the subunits of vascular Nox2, thus reducing its activity [153]. HNO is also known to potentiate heme oxygenase-1 mRNA and protein expression leading to a significant elevation in its antioxidant and cytoprotective activities. It should also be noted that HNO, by downregulating O_2_^−^ production, can increase the bioavailability of NO in oxidative stress. Impaired NO bioavailability is one of the most deleterious effect of aging on vascular well-being. Thus, HNO helps in maintaining proper functioning of CVS.

**Figure 6:**
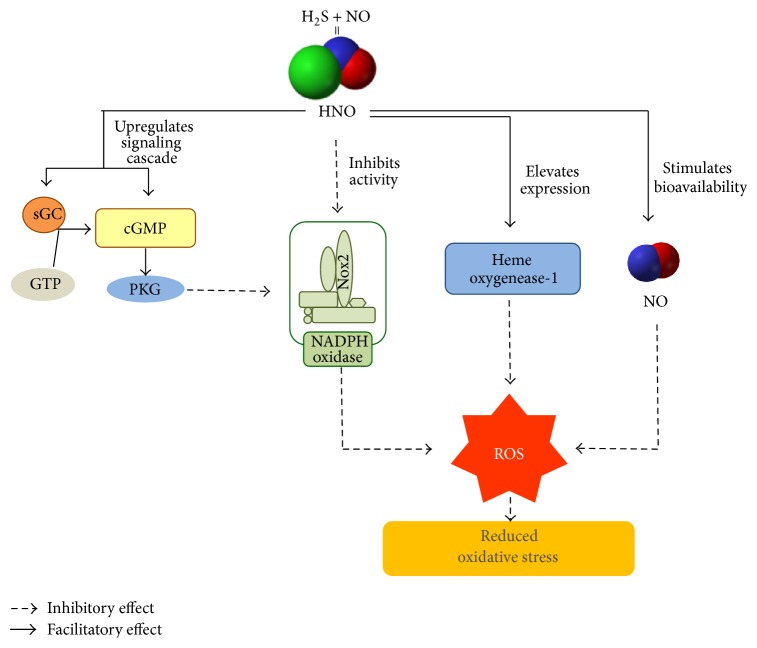
Role of H_2_S-NO interaction in oxidative stress in CVS. The primary target of action of HNO is NADPH oxidase, which is the main culprit enzyme for endogenous synthesis of ROS in aging CVS. HNO can inhibit its activity in both sGC-cGMP dependent and independent ways. HNO is also known to strengthen anti-inflammatory response by elevating heme oxygenase-1 expression. HNO increases the diminished bioavailability of NO in oxidative stress, resulting in proper functioning of heart and blood vessels.

Both H_2_S and NO have been shown to exhibit beneficial effect against oxidative stress in many biological systems including CVS. In last few years, the role of intermediate products released during the interaction between H_2_S and NO has also been studied in oxidative stress pathology (Figure 6). Now it is generally agreed that HNO has significant potential to function as an antioxidant and hence further investigation is necessary to explore its prospective therapeutic benefits.

### 6.1. Perspectives

In recent few years, a few research groups have demonstrated the formation of novel intermediate species during the reaction between H_2_S and NO. In the initial work, the mixture of various NO donors and H_2_S generated an intermediate formation with general properties similar to an S-nitrosothiol. Later, HSNO (thionitrous acid) was considered as the most likely S-nitrosothiol candidate [154]. Shortly after that discovery, a few of reports suggested HNO generation from the reaction between NO and H_2_S donors [49, 75]. The endogenous production of HNO is also speculated, and lots of efforts have been put in developing reliable HNO detection methods in order to understand endogenous HNO generation. Several approaches including electrochemical analysis [155], high-performance liquid chromatography [156], and mass spectrometry [157] have been used to detect HNO in various biological samples. However, these methods either lacked sensitivity or specificity towards endogenously generated HNO. Hence, novel HNO detection approaches including Cu^2+^-medicated fluorescent probes and HNO-specific electrodes were adopted [155, 158]. Recently, a novel coumarin-based fluorescent probe, P-CM, was developed. It has been applied for selective quantitative detection of HNO in bovine serum samples [159]. Jing et al. successfully detected lysosomal HNO in cell system (RAW 264.7 macrophage cells) and* in vivo* using a newly synthesized a near-infrared fluorescent probe Lyso-JN [160].

It is speculated that HNO is produced endogenously in low concentration and perhaps insufficient to elicit any significant physiological effect [49]. The mechanisms proposed are still under investigation and include NO reduction [161], reaction of S-nitrosothiol with thiols [162], and reaction catalyzed by NOS. HNO is biochemically different from NO and H_2_S and is known to show increased cardiac tissue contractility [163]. Furthermore, studies have implied that endogenous HNO production is elevated during pathological situations such as inflammation [7, 164]. Thus HNO and its derivatives are rapidly drawing attention as a potential pharmacological target in treatment of congestive cardiac failure [165] and acute heart failure [166].

However even with the favorable results, there are some concerns raised about the physiological relevance of these studies. For one thing, all these studies have utilized the supraphysiological concentrations of NO and H_2_S. Furthermore, the use of exogenously added NO donors, instead of stimulating endogenous source of NO, are also debatable. For example, SNP does not release NO spontaneously and thus fails to mimic endogenous production and response of NO [167]. The known reactivity of SNP and H_2_S further complicates the matter as it might generate products different from those generated in biological reaction between H_2_S and NO [47, 49, 154].

Although additional investigations are warranted to establish the exact mechanisms of action of H_2_S and NO, the importance and necessity of these two functional molecules in regulation of mammalian cardiovascular system are beyond debate. Our knowledge on these two gasotransmitters is expanding continuously and it is now evident that interaction between their molecular pathways is increasingly investigated as the future direction for the research in the area of gasotransmitters.
